# Emergence from General Anaesthesia: Can We Discriminate between Emergence Delirium and Postoperative Pain?

**DOI:** 10.3390/jpm13030435

**Published:** 2023-02-28

**Authors:** Marta Somaini, Thomas Engelhardt, Pablo Ingelmo

**Affiliations:** 1 Department of Anesthesia, ASST Grande Ospedale Metropolitano Niguarda, 20162 Milano, Italy; 2 Advanced Course Teacher Scuola di Specialità Anestesia e Rianimazione, Università degli Studi di Milano Bicocca, 20126 Milano, Italy; 3 Department of Anesthesia, Montreal Children’s Hospital, McGill University, Montreal, QC H4A3J1, Canada; 4 Edwards Family Interdisciplinary Center for Complex Pain, Montreal Children’s Hospital, Montreal, QC H4A3J1, Canada; 5 Research Institute, McGill University Health Center, Montreal, QC H4A3J1, Canada; 6 Alan Edwards Center for Research on Pain, McGill University, Montreal, QC H3A2B4, Canada

**Keywords:** emergence delirium, postoperative pain, postoperative care, general anaesthesia, postoperative complication, behavioral observation techniques

## Abstract

Unsettled behaviors characterize the early phase after general anaesthesia in the pediatric population in up to 80% of cases. Emergence delirium (ED) and acute pain are the two most relevant sources of this phenomenon. Research and clinical guidelines are difficult to implement due to the variability of the definition of unsettled behavior and measurement of the different components. The most probable incidence of ED is between 10% and 20%, and the potential risk factors could be summarized as young age, male gender, preoperative anxiety, baseline sleep-disordered breathing, volatile anaesthesia and ENT or ophthalmologic surgery. Self-reporting behavioral and observational scales are unable to reliably differentiate between ED and pain in a child who is not fully awake, making correct treatment choices difficult. This may lead to an undertreatment of pain in agitated children or to the overuse of opioids for self-limiting ED. This paper considers the current knowledge on the identification and treatment of ED and pain and provides a pragmatic approach for daily practice.

## 1. Introduction

Up to 80% of children can experience unsettled behaviors after awakening from general anaesthesia. Smessaert et al. described the concept of ‘mode of recovery’ in 1960. ‘Emergence Delirium’ (ED) was first defined one year later [1]. Several terms have been used since to describe the behaviors during the early phases of awakening, including early negative postoperative behavior, emergence agitation (EA), ‘emergence excitement’ and ‘maladaptive postoperative behaviour’ [1,2]. Frequently, these terms are used interchangeably, but they refer to different aspects and different etiology [3].

Emergence agitation and emergence excitement are used to describe more general conditions of ‘unpleasant state of extreme arousal’ where the child could experience pain, hunger, thirst or fear due to the absence of primary caregivers or unfamiliar surroundings [1]. Children are described as restless and presenting mental distress in the recovery room.

The *Diagnostic and Statistical Manual of Mental Disorders, Fifth Edition* (DSM-V) defines delirium as a state where the person has disturbances in attention and awareness (reduced ability to direct, focus, sustain and shift attention) associated with changes in cognition (disorientation, language disturbance) and perceptual disturbance. The core behaviors after awakening included eyes being averted, staring or closed, non-purposeful movements, non-responsivity, kicking, being inconsolable, not been able to make eye contact and being unaware of the surroundings. Up to 25% of children having ED are quiet, confused and disorientated. They do not make eye contact with the caregiver and may not be aware of their surroundings. These children would only make minimal movements when awake, are non-communicative and do not respond to social interactions. Described as hypoactive delirium, this behavior is less commonly recognized during emergence from general anaesthesia [4].

Emergence delirium may not be just an immediate recovery room problem [5]. One in four children who experienced ED presented negative behavior one week after surgery. Children with ED had higher risk of developing separation anxiety, apathy and sleep and eating disorders. They also experienced nightmares, woke up crying, had temper tantrums and developed new-onset enuresis [5,6,7].

Pain is defined as “An unpleasant sensory and emotional experience associated with, or resembling that associated with, actual or potential tissue damage”. Verbal description is only one of several behaviors to express pain. The inability to communicate does not negate the possibility that a human experiences pain. (https://www.iasp-pain.org/publications/iasp-news/iasp-announces-revised-definition-of-pain) A child in pain after surgery may scream, cry, touch the surgical site, have an abnormal facial expression or be inconsolable [5].

Pain and ED are the most relevant unsettled behaviors during the early phase after general anaesthesia. However, discrimination between different unsettled behaviors during awakening from general anaesthesia is still a challenge in daily practice [7,8]. Behavioral scales are unable to differentiate between ED and pain in a child who is not fully awake. Therefore, making the correct treatment choices is difficult. This may lead to an undertreatment of pain or to the overuse of opioids to treat ED [3,5].

In this review we consider the current knowledge for the identification and treatment of ED and pain and provide a pragmatic approach for the recovery room. 

## 2. Evaluation Tools and Scoring Systems

A large number of scoring systems and observational scales are currently used in clinical practice to evaluate unsettled behaviors after surgery. The Paediatric Anaesthesia Emergence Delirium scale (PAED scale) developed by Sickich and Lerman in 2004 is as of today the only one validated that allows the identification of ED [8]. The observational scales assessing EA, such as Cohen, Watcha and Cravero scale, primarily assess emotional distress and psychomotor agitation, which are characteristic of pain rather than diagnostic of delirium. The behaviors assessed in these scales in fact overlap with the scales validated to measure pain, such as the Faces, Legs, Activity, Cry and Consolability (FLACC) scale, the Children’s and Infants’ Postoperative Pain Scale (CHIPPS), or the Children’s Hospital of Eastern Ontario Pain Scale (CHEOPS) [1]. This overlapping among the scales could in part explain the efficacy of analgesics in the prevention and treatment of EA described in different clinical settings. Because most scales assessing EA have not been psychometrically tested and are susceptible to observers’ subjectivity, we will not further consider them in this review. 

## 3. Emergence Delirium

ED is a disturbance in attention and awareness associated with changes in cognition and perceptual disturbance. The incidence of ED is highly variable in the literature, with the most frequently reported incidences in young children ranging between 10% and 20% [1,9,10,11]. Potential risk factors could be summarized as young age (less than 6 years), male gender, preoperative anxiety, baseline sleep-disordered breathing [12], volatile anaesthesia and ENT or ophthalmologic surgery. ED occurs during the first 20 min after spontaneous awakening and almost always within 5 min after awakening; however, has been reported as late as 45 min after awakening from general anaesthesia. ED is self-limiting in almost all cases and never restarts in the same child [9,13,14,15]. The incidences of ED and pain have different trends during awakening from general anaesthesia, indicating that ED and pain are separate entities [8]. Clinicians should be aware that almost 15% of children can experience ED and pain at the same time, making the diagnosis and maybe the management of the former more challenging [16].

### 3.1. Common Features in Emergence Delirium

The PAED scale [8] includes two groups of main domains; the first group evaluates consciousness (‘Eye contact’ and ‘Awareness of the surroundings’) and cognition (‘Purposeful actions’). The second group, including ‘Restlessness’ and ‘Inconsolability’, assesses disturbances in psychomotor behavior and emotion. The categories ‘No eye contact’ and ‘No awareness of surroundings’ are unique to the PAED scale and perfectly reflect the definition of delirium. They are considered the most important items for the identification of ED [1,8].

The sum of the values of ‘Eye contact’, ‘Awareness of the surroundings’ and ‘Purposeful actions’ of the PAED scale has been described as a delirium-specific score (ED1). Conversely, the sum of the scores of ‘Restlessness’ and ‘Inconsolability’ of the PAED scale is used to identify a ‘non-specific delirium score’ (ED2) [14,16]. ED1 scores of 9 or more points were strongly correlated with ED episodes in the early phases of awakening [14,15,16]. The association of ‘No eye contact’ and ‘No awareness of surroundings’ was associated with a 99% sensitivity and 63% specificity of identified ED during the first 15 min after awakening from general anaesthesia. On the other hand, the association of ‘Abnormal facial expression’, ‘Crying’ and ‘Inconsolability’ resulted in 93% sensitivity and 82% specificity for detecting pain during the early postoperative period [15].

Hypoactive delirium can be assessed using the Cornell Assessment of Pediatric Delirium (CAP-D), an adaptation of the PAED scale. The CAP-D adds the following items to assess hypoactive delirium:

1. Does the child communicate needs and wants?
2. Is the child underactive—very little movement while awake?
3. Does it take the child a long time to respond to interactions?

### 3.2. The Difficulty of Differentiating between ED and Postoperative Pain

There is a significant overlap in the components of the PAED scale and of those included in the scales evaluating pain in young children [1,7,15]. For example, both the PAED and FLACC scales include ‘Consolability’ and ‘Purposeful action’. An evaluation of postoperative behavior only based on ‘Crying’ or ‘Facial expression’ in combination with ‘motor restlessness’ may result in a simultaneous diagnosis of pain and ED even in the absence of nociceptive stimulation or pain [1,15]. To complicate matters further, when evaluating children with the PAED and FLACC scales during the first 15 min after general anaesthesia for an MRI scan, children experiencing ED were found to be 4 times more likely to also exhibit pain behavior [17].

The FLACC scale is a reliable observational tool to assess pain in fully awake young children [18]. However, the FLACC scale may mislabel ED as pain during the first minutes after awakening when the child is not yet completely awake. In other words, in daily practice we are able identify ED episodes in children recovering from general anaesthesia, but we are unable to diagnose pain in children who are not fully awake (first 5–10 min after spontaneous awakening).

### 3.3. Prevention of Unsettled Behavior after General Anaesthesia

The impact of preoperative interventions on the development of ED remains undetermined, mainly due to the difficulty of the diagnosis in the post-anaesthesia period. However, pharmacological and non-pharmacological strategies used in the preoperative setting to reduce both patient and parental preoperative anxiety have shown effectiveness in reducing the incidence of unpleasant behaviors.

The risk of postoperative negative behavior changes is increased three to four times in children who experience preoperative anxiety [6,18]. Negative behavior seems not to be related with the intensity of pain, the type of surgery, the patient’s age or the use of premedication.

Clinicians should consider including tools to identify anxiety traits before surgery to improve the perioperative management of their patients. The modified Yale Preoperative Anxiety Scale (m-YPAS) is too complex to be used in a busy clinical practice. The State-Trait Anxiety Inventory for Children (STAIC) is an alternative predictor of anxiety and can be administered upon hospital arrival [6,19]. Tablet-based interactive distraction (TBID) demonstrated improved baseline anxiety, tolerance of mask induction and lower rates of ED at 15 min postemergence compared with benzodiazepine [20].

Preoperative administration of intranasal dexmedetomidine (1–2 mcg/kg) at least 20 min prior to induction was associated with less preoperative anxiety and ED [21]. Preoperative oral dexmedetomidine, compared to oral benzodiazepine, was associated with significantly lower PAED scores (*p* < 0.05) and ED rates (0% in Dex group, 19% in Midaz group (*p* = 0.01)) [22].

The Paediatric Anaesthesia Behaviour score can quantify the degree of anxiety during the induction of anaesthesia [23].

It identifies children who are distressed during the induction of anaesthesia and allows three distinctive scenarios:

1. Happy—Calm and controlled. Compliant with induction.
2. Sad—Tearful and/or withdrawn but compliant with induction.
3. Mad—Loud vocal resistance (screaming or shouting) and/or physical resistance to induction requiring physical restraint by staff and/or parents.

A high Paediatric Anaesthesia Behaviour score during induction can usefully predict the incidence and intensity of ED and correlates well with m-YPAS score [5,23].

A high Paediatric Anaesthesia Behaviour score may prompt clinicians to tailor their intraoperative plan using preventive measures to reduce emergence delirium. The uses of regional anaesthesia, propofol infusions, and intraoperative alpha-2 agonists were associated with a reduction in the incidence of unsettled behavior after surgery [3,7].

### 3.4. Management of Unsettled Behavior after General Anaesthesia

**Figure 1:**
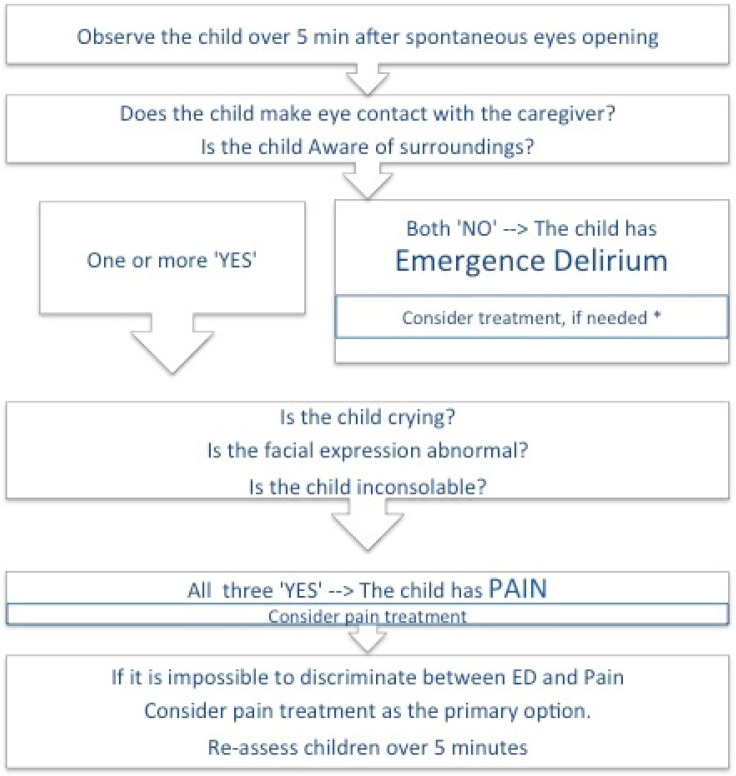
An algorithm to discriminate between ED and pain using five questions.

The treatment of unsettled behaviors after general anaesthesia should be according to the diagnosis made and according to the severity and duration of the symptoms [10]. Somaini et al. proposed an algorithm to discriminate between ED and pain using five questions (Figure 1) [15]. 

If the child has ‘no eye contact’ and is ‘not aware of surroundings’, ED should be suspected. [8,15] On the basis of the severity of the symptoms, the child can either be treated with general comfort measures (pacifier, sucrose, milk/apple juice) or by medication, such as alpha-2 agonists (dexmedetomidine 0.5 mcg/Kg IV) or propofol (1 mg/kg IV) [10,13,15,24].

The incidence and the course of ED are not affected by parental presence, so it is essential explain the phenomenon and reassure parents that ED is self-limiting and that their child will return to normal behavior within 15 min regardless of treatment [10]. However, parents are naturally concerned about the impact of surgery and anaesthesia, and they need help. A child having emergence delirium in the recovery room is often an incomprehensible scenario for the parents. They may feel powerless or guilty and experience fear and insecurity. Adequate and timely information as well as staff members being available to help them during the wake-up period may relieve this suffering [5,25].

If ED is excluded, acute pain should be considered as the source of the unpleasant behavior and treated promptly (i.e., fentanyl 1 mcg/kg IV). None of the observational scales validated to assess pain in awake children will be helpful in identifying acute pain in the first 10–15 min after general anaesthesia. In this scenario, if the child has an ‘Abnormal facial expression’, is ‘Crying’ and is ‘Inconsolable’, pain must be considered and should be treated according to locally established protocols [10,13,15,26].

If the cause remains unclear, apple juice, breastfeeding, general comforting and reassessment within 5 min may help to identify the underlying problem [10,13,15].

Of note, antipsychotics do not have an indication for use in children with delirium, and attention to possible side effects such as prolonged QTc are necessary if such use is deemed appropriate [26].

## 4. Conclusions and Key Points

During awakening from general anaesthesia, a large number of young children may present unpleasant behavior, primarily pain, ED or both. An accurate assessment is the key to success in the management of such conditions. The identification of the etiology is clinically relevant in order to provide effective analgesia to children in pain while preventing inappropriate use of opioids in children with ED.

1. Evaluate the presence of preoperative anxiety and unsettled behaviors during induction of anaesthesia.
2. Tailor the anaesthesia plan to prevent unsettled behaviors after general anaesthesia.
3. If the child has ‘No eye contact’ and is ‘Not aware of surroundings’, we should suspect and treat ED.
4. If the child has an ‘Abnormal facial expression’, is ‘Crying’ and is ‘Inconsolable’, the child is likely to be in pain.
5. If the cause of the unpleasant behavior is unclear, providing general comfort, offering something to drink and reassessing the patient within 5 min is required until the underlying problem is identified.
6. Support the parents of children displaying unsettled behaviors after anaesthesia. The problem may continue after discharge.

## Data Availability

Not applicable.
